# Supplementary material for: Effects of Disruption of Five FUM Genes on Fumonisin Biosynthesis and Pathogenicity in Fusarium proliferatum
Source: Toxins (Basel). 2019 Jun 7;11(6):327. doi: 10.3390/toxins11060327 (PMC6628412; doi:10.3390/toxins11060327)
Supplement: Supplementary file 1 [file toxins-11-00327-s001.pdf]

Supplementary Materials: Effects of Disruption of Five *FUM* Genes on Fumonisin Biosynthesis and Pathogenicity in *Fusarium proliferatum*

Lei Sun, Xu Chen, Jian Gao, Yuan Zhao , Lianmeng Liu, Yuxuan Hou, Ling Wang and Shiwen Huang

Table S1. Primers used in our experiments.

Primer Name	Sequence	Product (bp)
FUM1-FD-F	CTATAGGGCGAATTGGGTACCTTGCTGGTGATCTTGCAGAG	1142
FUM1-FD-R	CATTCATTGTTGACCTCCACTAACGAGTATAAGGTTACGGTGGAT	
FUM1-BK-F	GGGCAAAGGAATAGAGTAGATGAGGAGATTCTCAACTCTGGCACT	1542
FUM1-BK-R	CGCTCTAGAACTAGTGGATCCGCTACAGACCTCTTGGGTGC	
FUM1-YZ-F	TCAGGCATTTCGTCAGTT	750
FUM1-YZ-R	GAAGGTCTCGTATTCGTCC	
FUM1-YZS-F	ATTGAGTCTCTGTTGGATGA	1912
FUM1-YZS-R	TAAGTCAGATTGAATCTGAA	
FUM21-FD-F	CTATAGGGCGAATTGGGTACCGGCATCACAATCTATTAGTTCCT	1223
FUM21-FD-R	AGGCATTCATTGTTGACCTCCACTAAGACTTCAGCAAGGTCCACA	
FUM21-BK-F	CGAGGGCAAAGGAATAGAGTAGATGGCTCTTGCGCTACGCTATAA	1356
FUM21-BK-R	TCCCCCGGGCTGCAGGAATTCAAACCTCCTGGTACGCCCT	
FUM21-YZ-F	TTTCCAGAGTCCATTCTTACAC	714
FUM21-YZ-R	CGCCATCTACGACCACAA	
FUM21-YZS-F	TAAATACTTTAAGTATTACT	1966
FUM21-YZS-R	TAAGTCAGATTGAATCTGAA	
FUM6-FD-F	GGTATCGATAAGCTTGATATCCTTGAGATTGGAAAAAGAGACT	1253
FUM6-FD-R	AGGCATTCATTGTTGACCTCCACTAATTCTGGGGGTAGCTATGTT	
FUM6-BK-F	CGAGGGCAAAGGAATAGAGTAGATGCGTTTGGGTTGGGGCTT	1421
FUM6-BK-R	AAAGCTGGAGCTCCACCGCGGATTGCAGGGAGATACCTTACA	
FUM6-YZ-F	TCCTTCTTGACCGCATAC	756
FUM6-YZ-R	GATTGTCACGCCTTTGTT	
FUM6-YZS-F	TTCAGATTCAATCTGACTTA	1962
FUM6-YZS-R	TAAGTCAGATTGAATCTGAA	
FUM8-FD-F	GGTATCGATAAGCTTGATATCTCGTCGCGGGTATGTAGTAGA	1407
FUM8-FD-R	AGGCATTCATTGTTGACCTCCACTAGGAGGTTGTTGTCACAGGTTC	
FUM8-BK-F	CGAGGGCAAAGGAATAGAGTAGATGAATGGGTGTCTGATGGCTTG	1235
FUM8-BK-R	AGAACTAGTGGATCCCCCGGGGGGAGACCTTGTTGACAATTTC	
FUM8-YZ-F	GTTCCTGTGGCAGATTAGA	834
FUM8-YZ-R	CCAACCTTTTAGCATTCC	
FUM8-YZS-F	AAAAGCCTTCAATTCAACA	1874
FUM8-YZS-R	TAAGTCAGATTGAATCTGAA	
FUM19-FD-F	CTATAGGGCGAATTGGGTACCGCTTGACTGGGAGGCACA	1547
FUM19-FD-R	AGGCATTCATTGTTGACCTCCACTATTATCGCAAGGGGCTCAC	

FUM19-BK-F	CGAGGGCAAAGGAATAGAGTAGATGAGTATGAGCGTGCGTTGAGA	1148
FUM19-BK-R	AGAACTAGTGGATCCCCCGGGCTTGTGGGTTAGTTTCGTGGT	
FUM19-YZ-F	CAAATTGCTCACAACCGAG	754
FUM19-YZ-R	ACCAACTTGCCCTACGCT	
FUM19-YZS-F	ATATTTTATAGTCTAACCT	2054
FUM19-YZS-R	TAAGTCAGATTGAATCTGAA	
HygR-F	TAGTGGAGGTCAACAATGAAT	1357
HygR-R	CATCTACTCTATTCCTTTGCC	
RT*FUM1*-F	CCAACTCTTCTTCCCTGCTA	119
RT*FUM1*-R	CACCCTCTACCTCCCACA	
RT*FUM6*-F	CTGGAAAGTATGCGGTCAA	101
RT*FUM6*-R	GCAGAACTCATCAGCGTCA	
RT*FUM8*-F	GCGGAACGAGAAATAGTGA	117
RT*FUM8*-R	TGCTGGGTTGAAAGGGAG	
RT*TUB*-F2	ACATCCAGACAGCCCTTTGTG	106
RT*TUB*-R2	AGTTTCCGATGAAGGTCGAAGA	

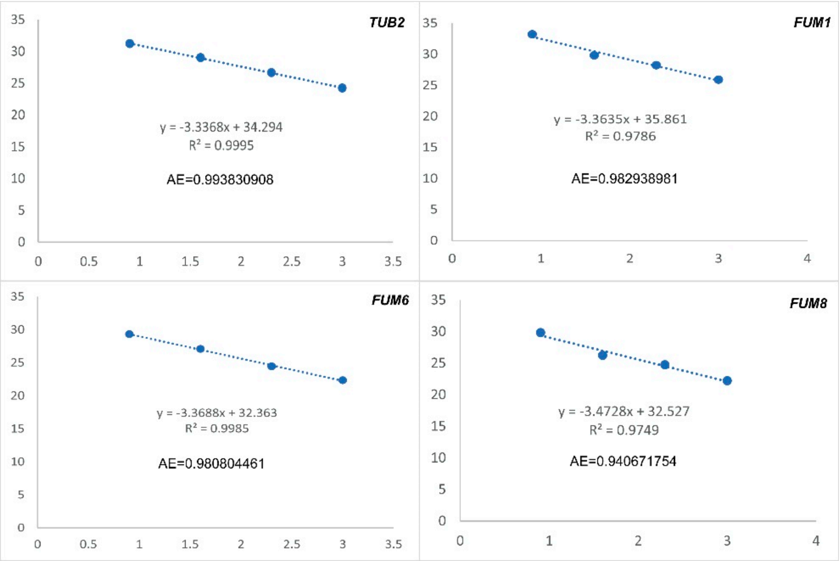

Figure S1. Amplification efficiencies (AEs) of qRT-PCR primer pairs.
